# Feasibility, acceptability, and limited efficacy of health system-led familial risk notification: protocol for a mixed-methods evaluation

**DOI:** 10.1186/s40814-022-01142-9

**Published:** 2022-08-09

**Authors:** Paula R. Blasi, Aaron Scrol, Melissa L. Anderson, Marlaine Figueroa Gray, Brooks Tiffany, Stephanie M. Fullerton, James D. Ralston, Kathleen A. Leppig, Nora B. Henrikson

**Affiliations:** 1 Kaiser Permanente Washington Health Research Institute, 1730 Minor Avenue, Suite 1600, Seattle, WA 98101 USA; 2 Department of Bioethics and Humanities, School of Medicine, University of Washington, Seattle, WA USA; 3 Kaiser Permanente Washington, Seattle, WA USA

**Keywords:** Genetic testing; Cascade testing; Lynch syndrome, Breast cancer, Ovarian cancer, Cancer risk

## Abstract

**Background:**

Genetic testing for pathogenic variants associated with hereditary breast and ovarian cancer risk can improve cancer outcomes through enhanced preventive care in both people with known variants and their biologic relatives. Cascade screening—the process of case-finding in relatives by notifying and inviting them to consider testing—currently relies on the patient to notify their own at-risk relatives. However, many of these relatives never learn they might be at risk. We developed and implemented a new health system-led familial genetic risk notification process where the care team offers to contact at-risk relatives directly. This protocol describes a study to assess the feasibility, acceptability, and limited efficacy of this intervention.

**Methods:**

This feasibility study will use a single-arm, nonrandomized, mixed-methods prospective design.

We will enroll two groups of participants: probands and relatives of probands. Eligible probands are currently enrolled Kaiser Permanente Washington (KPWA) members with an upcoming appointment for pre-test genetic counseling for hereditary Lynch syndrome, breast, or ovarian cancer. Eligible relatives, who do not have to be KPWA members, are first-and second-degree relatives of probands. During the appointment with the proband, the genetic counselor will determine whether the proband is appropriate for genetic testing and if so, which relatives might benefit from cascade testing. The genetic counselor then will offer to contact any or all identified relatives directly to discuss genetic risk and testing. The primary outcome of this study is the feasibility of the implemented familial notification process, which we will measure using quantitative and qualitative data on intervention reach, intervention acceptability, and limited efficacy. Analyses will be primarily descriptive and exploratory, with the intent of preparing for a future, larger trial of direct contact interventions.

**Discussion:**

Our findings will provide new, foundational evidence for the creation of US-based familial notification systems that directly address logistical and ethical challenges while prioritizing the preferences of patients and families.

**Supplementary Information:**

The online version contains supplementary material available at 10.1186/s40814-022-01142-9.

## Background

Certain genetic conditions run in families and increase the risk of adverse health outcomes. For example, hereditary breast and ovarian cancer syndrome (caused by variants in the *BRCA1* or *BRCA2* genes) and Lynch syndrome (caused by variants in the *MLH1*, *MSH2*, *MSH6*, or *PMS2* genes) increase the risk for colorectal, endometrial, ovarian, and other cancers. The U.S. Centers for Disease Control and Prevention classifies both conditions as Tier 1 genomics applications because testing for variants in these genes has significant potential for a positive impact on public health [1].

When genetic testing finds a pathogenic variant in these genes in one individual (a proband), there are immediate implications for the proband’s biological relatives, who may carry the same variant and be at increased risk of cancer. Current standard of care recommends that relatives of these probands be notified and referred for genetic testing. If the relatives carry the same pathogenic variant, they may undergo more frequent cancer screenings or even prophylactic surgery. Enhanced screenings and preventive surgery can lead to early detection or prevention of cancer, thereby improving health outcomes.

Currently, in the USA, patients are responsible for notifying their own family members about relevant genetic test results, and relatives are responsible for initiating their own genetic testing or follow-up care. However, many patients with pathogenic variants do not disclose their test results to all relatives who might be at risk. For example, a study [2] at Kaiser Permanente Washington (KPWA) found two of three patients with actionable genetic test results reported only partial disclosure to family members. Incomplete disclosure means that some relatives never learn they might be at increased risk and never receive genetic testing themselves. Other studies have found only about half of relatives of patients with Lynch syndrome ultimately receive genetic testing [3], and about 20–30% of eligible relatives receive *BRCA* testing [4].

Cascade testing is the process of case-finding within families by notifying at-risk relatives and inviting them to consider genetic testing. Studies outside of the USA have shown that health system-led direct contact interventions are acceptable to patients and families and notify more relatives than patient-led contact alone [5–9].

Although US clinicians are under no legal obligation to notify relatives about genetic disease risks, clinicians often report feeling a personal sense of duty to their patients’ relatives. In a survey of US genetic counselors [10], 46% reported cases in which a patient refused to inform a relative of their risk, and 63% agreed with the idea that genetic counselors have an obligation to notify at-risk relatives. In a follow-up study [11], 69% of medical geneticists reported feeling a duty to notify their patients’ relatives of potential genetic risks.

### Preliminary studies

To explore the feasibility of health system-led familial genetic risk notification in a US setting, we conducted two preliminary studies. In the first study [12], we interviewed 20 patients who were awaiting genetic test results. These interviews explored participants’ views on familial notification related to hypothetical test results for variants unrelated to their own indication for testing. We found that most participants generally supported the idea of health system-led direct outreach, particularly for variants with high clinical urgency. However, participants also noted a desire to alert their relatives first or get their relatives’ permission before the health system reached out, and some patients voiced concerns related to family relationships, relatives’ privacy, and insurance discrimination. These 20 participants named 535 first- or second-degree relatives and identified 76 of these as current or former KPWA members. This suggests each KPWA proband may have at least 2–3 relatives who may be available for notification about genetic testing and follow-up and who are also KPWA members, as well as potentially more who receive care outside of KPWA.

For our second preliminary study [13], we pursued a human-centered design approach. We invited probands (*n*=12) and relatives (*n*=46) to co-design a new health system-led familial notification process. In the first round, focus group participants discussed the current state of familial genetic risk notification and used storyboards to design a new health system-led process. In the second round, a different group of participants viewed a video version of the newly created process and answered questions about its acceptability and value. After conducting a thematic analysis and confirmatory analysis of our focus group and interview data, we identified four major themes related to participants’ preferences on direct contact interventions as well as six design requirements for implementing such an intervention (Table 1). Using the findings from this study, we developed a new familial genetic risk notification process in our health system; we are evaluating this process with the current study, called the Lynx study.

**Table 1:** Participant preferences and design requirements for a health system-led direct contact for cascade screening [12]

Themes related to participant preferences	Design requirements
• Patients generally support direct contact but have concerns about who is responsible for notifying relatives and how privacy will be protected • The main rationale for direct contact programs is the potential health benefit of notifying relatives of actionable genetic risk • Direct contact should be a program rather than an individual provider’s responsibility • Direct contact is a complement to, not replacement of, patient-led notification	Health systems should: • Obtain patient consent before contacting relatives • Employ multiple attempts and communication channels to reach relatives • Provide relatives with the opportunity to decide which information they want to receive and how to act on it • Clearly state their reason for contacting relatives and offer information on potential risks and related diseases • Make clear recommendations for genetic testing and follow-up steps • Continue providing patients with support and written resources to share with their relatives

### Objectives

The primary objective of the Lynx study is to assess the feasibility, acceptability, and limited efficacy of health system-led direct contact of relatives. We will use a mixed methods approach to evaluate participants’ experience of the intervention and quantify aspects of its potential impact.

## Methods/design

### Study design

The Lynx study is a feasibility study with a single-arm, nonrandomized, mixed-methods prospective design.

### Setting

The Lynx study will take place at Kaiser Permanente Washington (KPWA), an integrated health care delivery system that provides care and coverage to more than 710,000 members in Washington state.

### Funding

The Lynx study is funded by the National Human Genome Research Institute (R01HG010144). The funding agency was not involved in the design of the study or in the writing of this manuscript.

### Participants

We will enroll two groups of participants: probands and relatives of enrolled probands. Eligible probands are currently enrolled KPWA members with an upcoming scheduled appointment with KPWA Genetic Services for pre-test genetic counseling for hereditary Lynch syndrome, breast, or ovarian cancer. Eligible relatives, who do not have to be KPWA members, are first-and second-degree relatives of enrolled probands. First-degree relatives include biological parents, siblings, and children; and second-degree relatives include biological grandparents, grandchildren, uncles, aunts, nephews, nieces, and half-siblings. Probands must identify the relatives and provide permission for the Lynx study to contact them, and the Lynx genetic counselor must identify the relatives as candidates for cascade testing. Both probands and relatives must be age 18 or older and able to complete the consent process and study surveys in English. We will exclude individuals currently receiving hospice care.

### Recruitment

We will use administrative data to identify potentially eligible probands with upcoming appointments. Each identified proband will receive a mailed study information sheet and letter inviting them to participate in the study, informing them that we will follow up by phone, and providing opt-out information. In the follow-up phone call, a study team member will explain the study and provide a paper or electronic consent form to those interested in participating.

During the appointment with participating probands, a KPWA genetic counselor will identify which relatives might benefit from cascade testing and offer to contact any or all of these relatives directly. For each relative for whom the proband requests this direct notification process, a study team member will mail a study information sheet and letter inviting the relative to participate in the study, informing them that we will follow up by phone, and providing opt-out information. In the follow-up phone call, a study team member will explain the study and provide a paper or electronic consent form to those interested.

As part of a follow-up survey conducted 6–8 weeks post-enrollment, we will invite probands and relatives to participate in additional semi-structured interviews about their experience with the intervention. Participants will receive a $20 incentive upon completion of the follow-up survey. We will contact interested participants to schedule an interview.

### Anticipated enrollment

We expect about 10 new patients per month will meet the proband eligibility criteria (outlined in the “Participants” section), for a total of approximately 120 potentially eligible patients during the 12-month intervention period. Since this is a pilot study, sample size calculations were not performed. A sample size of 120 potentially eligible patients will allow testing of important study processes such as eligibility assessment and patient contact, as well as evaluation of key study outcomes related to reach (study participation rates) and intervention acceptability. The pilot study will recruit all potentially eligible participants identified over a 12-month period, thus the total number identified combined with the recruitment rate will inform feasibility of a larger trial to evaluate intervention efficacy. Assuming an enrollment rate of 50%, we expect about 60 probands to enroll in the study. The results from our first preliminary study suggest that each proband will have about 3 first- or second-degree relatives eligible for cascade screening; therefore, we expect to identify up to 180 potentially eligible relatives (3 relatives for each of the 60 enrolled probands). Assuming an enrollment rate of 50%, we expect about 90 relatives to enroll in the study. The expected number of relatives is based on the preliminary study, which identified relatives who were KPWA members. To maximize our understanding of the feasibility and acceptability of the program, non-KPWA members also are eligible to enroll in the Lynx study.

### Intervention

**Fig. 1:**
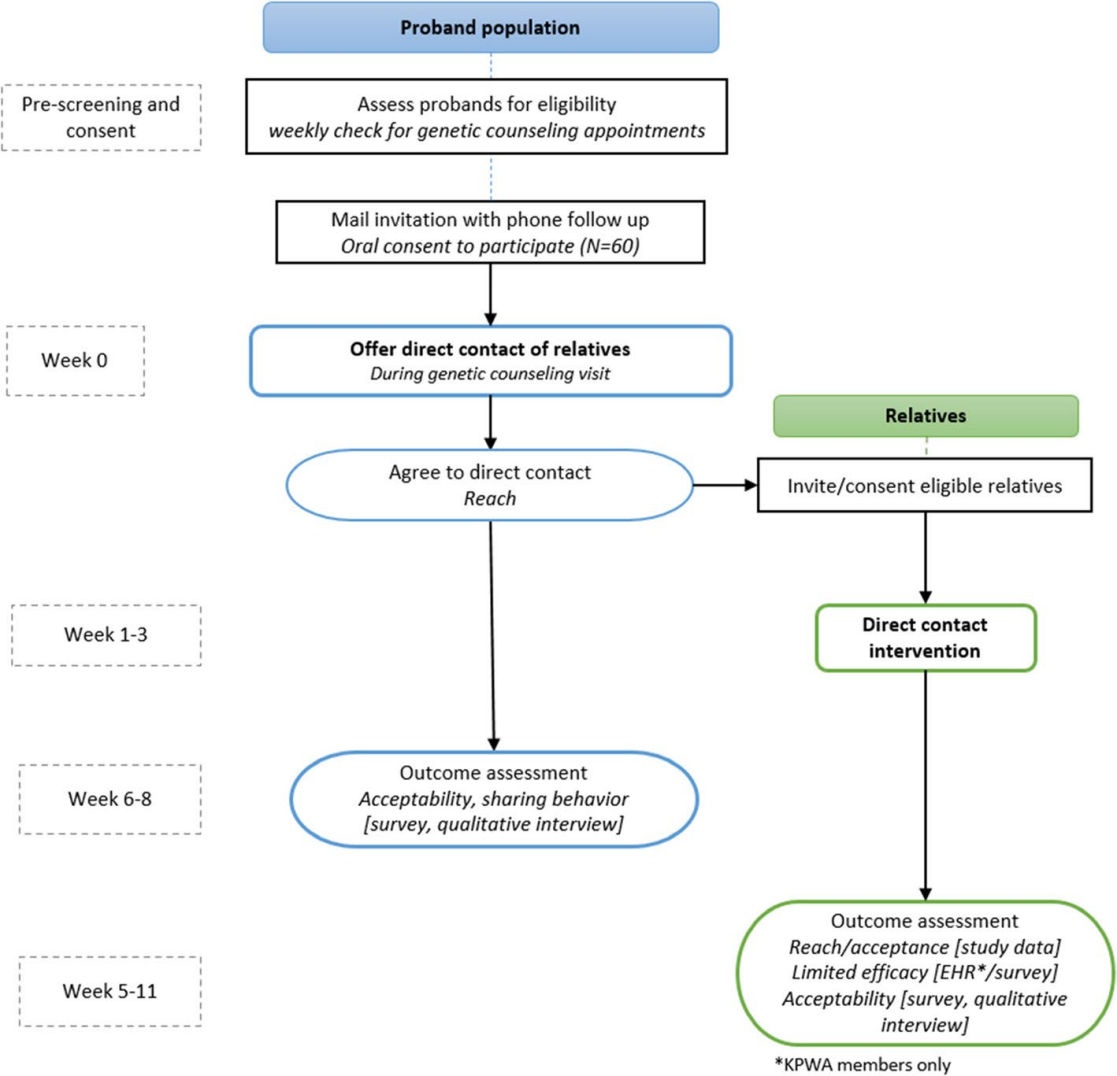
Study Flow

We designed our intervention to serve as a supplement to usual care received by KPWA genetic counseling patients. Figure 1 illustrates our expected study flow.

Study staff will send a secure email notification to KPWA Genetic Services about upcoming genetic counseling appointments with any probands who have consented to participate in the Lynx study. During the appointment, the genetic counselor will assess personal and family cancer history and make a recommendation about genetic testing following Kaiser Permanente (KP) regional care guidelines. If the proband decides to pursue testing, the counselor will complete a three-generation family pedigree to identify relatives of the proband who might benefit from cascade testing. Since consent occurs before the genetic counseling appointment, if the proband and counselor decide not to pursue testing, the proband is still eligible to continue with all study data collection activities, including their views on the general acceptability of direct contact programs; however, relative recruitment and notification is not applicable for these probands.

For probands who receive genetic testing, the genetic counselor will provide several options for notifying the proband’s identified relatives that they might benefit from genetic testing. As part of usual care, the proband can decide not to notify relatives, or the genetic counselor can provide the proband with a letter to share with relatives. As part of the Lynx intervention, the genetic counselor also will offer to contact any or all identified relatives directly. For each relative for whom the proband has requested the direct notification process, the genetic counselor will ask the proband to provide the relative’s name, relationship to proband, and contact information, and confirm that the relative speaks English. The genetic counselor also will ask whether the proband would prefer to have their name mentioned in the relative’s study invitation letter or to be referred to simply as “your relative.”

A study team member then will contact the identified relatives and ask if they are interested in participating in the study. If a relative declines to participate, the genetic counselor will inform the proband, who can decide whether to notify the relative themselves. If a relative agrees to participate and completes the consent process, the genetic counselor will contact them by phone. During the phone call, the genetic counselor will discuss the proband’s test results. If the proband has asked to remain anonymous, the genetic counselor will explain that the proband’s name is being withheld at the proband’s request. After discussing the proband’s test results, the genetic counselor will recommend genetic counseling or testing based on KP care guidelines. For relatives who are KP members, the genetic counselor will provide a referral to KP Genetic Services for counseling and testing. For non-KP members, the genetic counselor will direct relatives to resources for pursuing genetic counseling and testing outside of KP.

### Outcomes

Outcomes of interest for this feasibility study are outcomes essential to planning a larger efficacy trial, including reach, acceptability, and limited efficacy of the direct contact intervention. We will measure these outcomes using quantitative and qualitative data collection methods.

### Measures

**Fig. 2:**
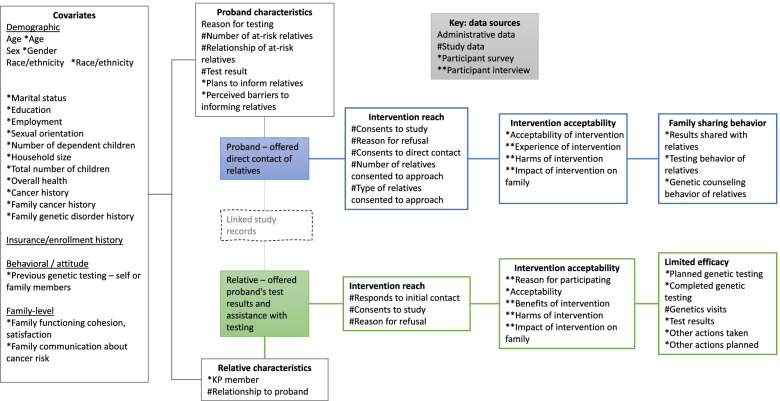
Conceptual data model

Our study will rely on data from electronic health records, genetic counseling visits, baseline and follow-up participant surveys, and qualitative interviews (Fig. 2; Additional file 1). Data on participant characteristics, including demographics, health history, family health history, previous genetic testing, and family dynamics will come from administrative data (probands) and the baseline survey (probands and relatives). Data on genetic test results and the number and relationship of at-risk relatives will come from genetic counseling visits.

To measure intervention reach, we will use data from genetic counseling visits to quantify the number of probands and relatives who respond to study recruitment, consent to participate, and reasons for refusal. Additional measures of intervention reach include the number of probands who consent to the direct contact process and the number and type of relatives who the proband identifies for direct contact.

We will measure intervention acceptability using both a follow-up survey conducted 6–8 weeks post-enrollment, and semi-structured interviews with a subset of participants who agree to participate. The follow-up survey (Additional file 1) includes 10 acceptability items created de novo for this study based on Sekhon et al.’s theoretical framework of acceptability [14, 15] and 5 items from Brehaut et al.’s decision regret scale [16] with wording adapted for clarity. For these items, response options are a five-point Likert scale ranging from “strongly disagree” to “strongly agree.” In addition, the guide for our semi-structured interviews (Additional file 1) includes questions related to reasons for participating, experience of the intervention, benefits of the intervention, negative effects of the intervention, ethical issues related to familial genetic risk notification, and alternatives to the intervention.

By limited efficacy, we mean this study will collect data necessary to inform potential intervention effect sizes to provide insight on the potential impact of the intervention for a future larger trial [17]. For a future randomized controlled trial, our primary outcome will be the proportion of at-risk relatives notified. This outcome reflects the primacy of autonomous decision-making of relatives in whether to pursue testing. For the present study (Lynx), we will measure this primary outcome and other measures of family sharing behavior using the follow-up survey. For both probands and relatives, measures of family sharing include questions about the types of relatives with whom the proband shared their genetic test results and the number and types of relatives who subsequently had genetic counseling and/or testing. For relatives only, additional measures include whether the relative has or intends to have genetic testing after talking with the Lynx genetic counselor, the relative’s genetic test results (if tested), and 8 items adapted from Seiffert et al. [18] about whether the relative took specific actions after talking with the Lynx genetic counselor, such as contacting their doctor or changing their diet or exercise habits. Secondary outcome measures include the number of relatives who attended appointments with KPWA’s clinical genetics service by 6–8 weeks post-intervention, as well as time to genetic testing.

In addition to the feasibility and secondary outcomes, we also will examine changes in family dynamics from pre- to post-intervention. Both the baseline and follow-up surveys include 6 items on family communication from Bowen et al. [19], as well as 7 items on family cohesion and 8 items on family satisfaction from Olson’s Family Adaptability and Cohesion Evaluation Scale (FACES) [20].

### Analysis

Analyses will be descriptive and exploratory, with the intent of preparing for a future, larger trial of direct contact interventions. As a feasibility study, the analytic plan includes descriptive analyses and select group comparisons to evaluate the intervention’s feasibility and acceptability, and to estimate the potential impact on study outcomes, including the proportion of relatives who seek referral to genetic counseling and complete genetic testing. For survey and administrative data, we will conduct bivariate descriptive analyses of all responses, with measures of differences between probands who did and did not use the direct contact intervention.

For qualitative interview data, we will conduct a thematic analysis of study transcripts, with identification of emerging and global themes across interviews, using thematic groups in our conceptual model as a basis (for example, overall acceptability of new process). We will then synthesize the qualitative and quantitative data according to domains shared across data collection methods using triangulation and joint displays.

To determine the feasibility for a future trial, we will assess both quantitative and qualitative findings of reach, acceptability, and limited efficacy. For quantitative assessment of reach, we will consider a threshold of a minimum 25% consent rate among eligible probands approached to indicate feasibility. For quantitative assessment of acceptability, our threshold will be 75% or more respondents endorse either “agree” or “strongly agree” to the following item from the follow-up survey: “I am comfortable with the idea of this study.” For limited efficacy, we will use the proportion of eligible relatives successfully offered risk information via direct contact. While we have no firm threshold for this outcome, we will use pilot study findings to plan sample size and population selection for the future trial.

Not reaching pre-established criteria for quantitative outcomes does not necessarily indicate unfeasibility but may instead indicate changes needed to the protocol. Therefore, for all feasibility outcomes, we will use triangulation to examine both the stated thresholds and findings from qualitative data (for example, perceived burden or benefits of the intervention and suggestions for improvement of the intervention workflow as reported in qualitative interviews) to make final decisions about the design of a future clinical trial.

### Data management

Data for the baseline and follow-up surveys will be collected and managed using REDCap software, and all participant research data will be collected and stored on a secure server at KPWA. These data will not include the participants’ contact or identifying information. Rather, individual participants and their research data will be identified by a unique study identification number. The study data entry and management systems will be secured and password-protected, and all study staff have received training in protecting the confidentiality of research participants. At the end of the study, all study databases will be de-identified and archived.

### Ethical approval and study monitoring

This study protocol was approved by the Kaiser Permanente Washington Institutional Review Board (KPWA IRB) on June 4, 2021. The team will perform internal quality management of study conduct, data collection, documentation, and completion, following a quality management plan. The KPWA IRB will review and monitor any protocol modifications, adverse events, and unintended effects of the intervention.

### Dissemination

This study will comply with the National Institutes of Health (NIH) Public Access Policy, which ensures that the public has access to the published results of NIH-funded research. It requires scientists to submit final peer-reviewed journal manuscripts that arise from NIH funds to the digital archive PubMed Central upon acceptance for publication. In addition, every attempt will be made to publish results in peer-reviewed journals. Data and consent materials from this study may be requested by contacting the study principal investigator (NBH).

## Discussion

The Lynx study is a novel mixed-methods study assessing the acceptability, reach, limited efficacy, and lived experience of a health system-led familial genetic risk notification process in a prospective sample of US patients receiving genetic counseling for hereditary cancer risk and their relatives. Uptake of cascade screening in families with known pathogenic variants associated with hereditary breast and ovarian cancer or Lynch syndrome continues to be suboptimal, due in part to incomplete notification of at-risk relatives. Approaches to support patient-led risk notification, the current US standard, may help to improve risk notification [21], subsequent uptake of cascade testing, and improved cancer outcomes [22]. A more disruptive and potentially more effective strategy to improve rates of relative notification involves direct contact of relatives by health systems; this is the intervention under exploration in the Lynx study.

Studies outside the US suggest health system-led direct contact is acceptable to patients and families and can increase the identification and follow-up of at-risk relatives. For example, a study [5] conducted in an Australian familial cancer service compared patient-led notification with a direct outreach intervention in which the cancer service mailed information directly to relatives to inform them of their risk and the availability of genetic testing. Another study [6] conducted in the Netherlands found that health system-led outreach about cascade screening for familial hypercholesterolemia was acceptable to relatives and led to high acceptance of screening. Other studies of health system-led direct contact have found similar results and acceptability levels among patients in Finland [7], the UK [8], and Sweden [9].

The Lynx study is one of the first studies to explore a health system-led direct contact intervention in a US setting. As a new, relatively untested strategy in US settings, it is important to understand in some detail how to design such a program; its acceptability particularly as it relates to patient and relative privacy; and its potential reach to at-risk relatives who would otherwise go un-notified. We have designed this study accordingly: we developed the intervention based on feedback from patients and relatives using human-centered design methods [13], which provided the design requirements that we followed in designing the intervention, including approaching direct contact as a complement, not a replacement of patient-led contact; obtaining patient consent before contacting relatives; obtaining relatives’ consent to be contacted as early as possible (before proband testing); and strategies and messages for contacting relatives (Table 1).

Further strengths of our study include our strong care delivery partnerships with medical genetics and genetic counseling; its setting in an integrated health system that ensures and provides prevention-focused medical care for multiple generations; and our use of multiple data sources—including EHR, genetic counseling notes, surveys, and qualitative interviews—to evaluate a variety of quantitative and qualitative outcomes. As such, the Lynx study will provide rich data that inform both the design of a future effectiveness trial and the lived experience of participants, which is critical in implementing a novel intervention.

In our design work, patients and relatives expressed strong preferences for approaching relatives well before proband testing occurs, to protect the privacy of both patients and relatives and to respect relatives’ right to decline to receive information. As such, we have intentionally designed the Lynx study to recruit potential probands before their genetic counseling appointment. However, this means that our sample sizes both of patients who decide to pursue genetic testing and of patients with pathogenic results are more limited than if we had focused only on recruiting people with pathogenic findings. We find this an acceptable tradeoff given the importance of privacy protections and relative autonomy, and this choice will allow us to estimate recruitment goals for a future larger trial.

Limitations of the study include its single site setting, which may limit generalizability to other sites. In addition, we are unable to systematically assess outcomes such as uptake of genetic testing and test results for relatives who receive care outside of KPWA. Another limitation is the potential for response bias; despite our focus on recruiting probands and relatives regardless of their choice to participate in direct contact, eligible participants’ choice to join the study may be related to their views about direct contact. However, we will leverage our qualitative interviews to collect rich narrative data about topics such as reasons for participating in the study; relatives’ interest or lack of interest in the study, family dynamics, family conversations about genetic testing, and actions taken after learning test results.

Findings of the Lynx study will provide new, foundational evidence that will inform the implementation of US-based familial notification systems; directly address logistical and privacy challenges while centering the preferences of patients and families; and provide critical data for design of a future randomized trial.

## Supplementary Information

**Additional file 1.** Lynx Protocol.

## Data Availability

The datasets that will be used and/or analyzed during the course of this study, as well as consent materials, are available from the corresponding author on reasonable request.

## Abbreviations

KPWA: Kaiser Permanente Washington
KP: Kaiser Permanente
