# Supplementary material for: Feasibility, acceptability, and limited efficacy of health system-led familial risk notification: protocol for a mixed-methods evaluation
Source: Pilot Feasibility Stud. 2022 Aug 9;8:174. doi: 10.1186/s40814-022-01142-9 (PMC9361690; doi:10.1186/s40814-022-01142-9)
Supplement: Supplementary file 1 — Additional file 1. Lynx Protocol. [file 40814_2022_1142_MOESM1_ESM.docx]

Additional File 1

[Table 1. Baseline and Participant Followup Surveys 2](#_Toc78545973)

[Table 2. Guide for Semi-Structured Interviews with Probands and Relatives 12](#_Toc78545974)

[References 15](#_Toc78545975)

# Table 1. Baseline and Participant Followup Surveys

| **Construct** | **Items**^a^ | **Response options** | **Included in Which Surveys**^b^ |
| --- | --- | --- | --- |
| General health | In general, would you say your health is: | Excellent / Very Good / Good / Fair / Poor | PB, RB |
| Cancer history | Have you ever been diagnosed with any type of cancer (1)? [if yes] what kind of cancer(s)? | No / Yes / Don’t know  [text for cancer type] | PB, RB |
| Family cancer history | Have any of your first-degree relatives (parents, brothers, sisters or children) ever been diagnosed with any type of cancer (1)? [if yes] how many first-degree relatives? | No / Yes / Don’t know  [text for number of relatives] | PB, RB |
| Family cancer history | Have any of your second-degree relatives (includes uncles, aunts, nephews, nieces, grandparents, grandchildren, half-siblings, and double cousins) ever been diagnosed with any type of cancer (1)?  [if yes] how many second-degree relatives? | No / Yes / Don’t know  [text for number of relatives] | PB, RB |
| Family genetic disorder | Before you joined this study, were you ever told by a doctor that you or a close family member has a genetic disorder? | Yes / No / Don’t know | PB, RB |
| Previous genetic testing: medical | Before this study, have you ever had genetic testing that was ordered by your doctor (1)? | Yes / No / Don’t know | PB, RB |
| Previous genetic testing: direct-to-consumer | Have you ever had genetic testing that you ordered yourself, such as from a company like 23andMe or Ancestry.com (1)? | Yes / No / Don’t know | PB, RB |
| Previous genetic testing: family | Before you joined this study, have any of your biological relatives ever had any type of genetic testing (2)? | Yes / No / Don’t know or decline | PB, RB |
| Family communication | Within the last year, how much have you spoken about cancer risk with each of the following family members (3)?   1. Your mother 2. Your father 3. Your sisters 4. Your brothers 5. Your children 6. Your grandchildren 7. Other (please describe) | Don't currently have this relative / not at all / a little / some / a lot  [text for other (please describe)] | PB, RB, PF, RF |
| Family intent to share | For each statement below, select one answer. Select N/A for not applicable if a statement does not apply to you.  If I decide to have genetic testing for cancer risk, I would share the results with (2):   1. My spouse or partner 2. My children 3. My mother 4. My father 5. My siblings 6. My friend 7. My primary care physician 8. Another health care provider (please describe): 9. i. Other (please describe): | Yes / No / Unsure or have not decided / Not applicable (N/A)  [text for (please describe)] | PB |
| Perceived barriers to informing relatives | Do you think any of the following will prevent you from discussing your results with one or more family members (4)?   1. Concern about upsetting relatives 2. Recalling painful memories 3. Difficult family relationships 4. Hard to talk about cancer risk 5. Age differences 6. Relatives live too far away 7. Lost touch with some relatives 8. Information not useful to relatives 9. Other (please describe) | Yes / No / Decline to answer | PB |
| Family cohesion | We would now like to get your thoughts on your family interactions. For each of these questions, please define “family members” as people who are biologically related to you (5).   1. Family members are involved in each other’s’ lives. 2. Family members feel very close to each other. 3. Family members are supportive of each other during difficult times. 4. Family members consult each other on important decisions. 5. Family members like to spend some of their free time with each other. 6. Although family members have individual interests, they still like to spend some of their time with each other. 7. Our family has a good balance of separateness and closeness. | Almost never / Once in a while / Sometimes / Frequently / Almost always | PB, RB, PF, RF |
| Family satisfaction | Again, for each of these questions, please define “family members” as people who are biologically related to you. How satisfied are you with (5):   1. The degree of closeness between family members. 2. Your family’s ability to cope with stress. 3. Your family’s ability to be flexible. 4. Your family’s ability to share positive experiences. 5. The quality of communication between family members. 6. The way problems are discussed. 7. The fairness of criticism in your family. 8. Family members concern for each other. | Very Satisfied / Somewhat Satisfied / Generally Satisfied / Very Satisfied / Extremely Satisfied | PB, RB, PF, RF |
| Open-ended | What else would you like us to know about your experience with genetic testing or talking with your family? | [Text] | PB |
| Open-ended | What else would you like us to know? | [Text] | RB, RF |
| Acceptability | For these questions, please consider “this study” to be when a genetic counselor, with a patient’s permission, contacts their relatives with health information that could benefit the relative.  Indicate how much you agree with the following statements (6, 7):   1. I am comfortable with the idea of this study. 2. Being in this study has been a good experience. 3. The surveys in this study took too much effort or time. 4. Talking with the genetic counselor for this study took too much effort or time. 5. Participating in this study made my life easier. 6. Participating in this study was worth the effort or time. 7. Being in this study helped me or my family. 8. My [relatives / I] only learned of [their/my] possible genetic risk because of this study. 9. This study will help me or my family member(s) manage our cancer risk. 10. This study will help me or my family member(s) in other ways. | Strongly Agree / Agree / Neutral / Disagree / Strongly Disagree | PF, RF |
| Decision Regret | Please tell us how you feel now about your choice whether to accept the genetic counselor’s offer to contact your relatives. For each statement below select one option (8).   1. It was the right decision 2. I regret the choice that was made 3. I would go for the same choice if I had to do it over again 4. The choice did me a lot of harm 5. The decision was a wise one | Strongly Agree / Agree / Neither Agree Nor Disagree / Disagree / Strongly Disagree | PF, RF |
| Family sharing | For each statement below, select one answer. Select N/A for not applicable if a statement does not apply to you.  When I received my genetic test results, I shared them with:   1. My spouse or partner 2. My children 3. My mother 4. My father 5. My siblings 6. My friend 7. My primary care physician 8. Another health care provider (please describe): 9. Other (please describe): | Yes / No / No but I plan to / Unsure or have not decided / Not applicable (N/A)  [text for “(please describe)”] | PF, RF |
| Family testing behavior | Has a first-degree relative (i.e., child, parent, sibling) had any type of genetic testing as a result of sharing your genetic test results with them (2)?   1. My children  IF YES: How many? 2. My mother 3. My father 4. My siblings  IF YES: How many? | Yes / No / Don’t know / Not applicable  [text for “How many?”] | PF, RF |
| Family genetic counseling behavior | Has a first-degree relative (i.e., child, parent, sibling) had genetic counseling since you shared your genetic test results with them?   1. My children  IF YES: How many? 2. My mother 3. My father 4. My siblings  IF YES: How many? | Yes / No / Don’t know / Not applicable  [text for “How many?”] | PF, RF |
| Relative test behavior | Did you have genetic testing after talking with the Lynx genetic counselor? | Yes / No / Don’t know | RF |
| Relative test plan | Do you plan to have genetic testing after talking with the Lynx genetic counselor? | Yes / No / Don’t know | RF |
| Relative other behavior | Which of the following did you do after talking with the Lynx genetic counselor? Select all that apply (9).   1. Spoke to my doctor 2. Spoke to another genetic counselor 3. Talked with my biological children about genetic testing 4. Talked with other biological family members about genetic testing 5. Talked with my family, friends, counselors, or religious advisors for support 6. Changed my diet or exercise habits 7. Done more of what I have always wanted to do 8. Looked up information about cancer risk 9. Other [open text] | Checkboxes  [text for “Other”] | RF |
| Test results: self-report | [if tested only] What did the doctor or genetic counselor tell you about your test results? | That I have a genetic variant that increases my cancer risk / That the results were normal or not concerning / That the results were not conclusive / I have not received my test results yet / Unsure | RF^c^ |
| Open-ended | What else would you like us to know about your experience with the study? | [Text] | PF |
| Age: self-report | What year were you born? | [xxxx] Year | RB |
| Gender identity: self-report | What is your gender? | Female / Male / Other | RB |
| Marital status | What is your current marital status (2)? | Married / Widowed / Divorced / Separated / Never married | PB, RB |
| Marital status | Are you currently living with a boyfriend/girlfriend or partner? | Yes / No | PB, RB |
| LGBT | Do you consider yourself to be gay, lesbian, bisexual, or transgender? | Yes / No / prefer not to answer | PB, RB |
| Race: self-report | What race(s) or ethnicity do you consider yourself to be [check all that apply] (1)? | White / Black or African American / American Indian or Alaska Native / Asian / Native Hawaiian and other Pacific Islander/ Other [specify] | PB, RB |
| Ethnicity: self-report | Do you consider yourself to be of Hispanic, Latino, or Spanish origin (1)? | Yes / No | PB, RB |
| Employment | What is your current work situation (Check all that apply) (1)? | Working / Temporarily laid off, sick leave or maternity leave / Looking for work, unemployed / Retired / Disabled, permanently or temporarily / Homemaker / Student / Other [please describe] | PB, RB |
| Education | What is the highest level of schooling you have completed [select one option] (1)? | 8th grade or less / Some high school l/ High school graduate or GED / Technical or trade school / Some college or a 2-year college degree / 4-year college graduate/ Postgraduate degree (Masters, PhD, MD, DVM, JD) | PB, RB |
| Household size | Including yourself, how many people currently live in your household (2)? | [x] people | PB, RB |
| Children - total | How many children (including both biological and non-biological) do you have (2)? | [x] children | PB, RB |
| Children - dependent | How many children under the age of 18 years currently live in your household (2)? | [x] children | PB, RB |

Legend:

^a^ Citations provided for survey items taken or adapted from other sources

^b^ PB = Proband baseline survey; RB = Relative baseline survey; PF = Proband followup survey; RF = Relative followup survey

^c^ Genetic test results collected from administrative data for probands

# Table 2. Guide for Semi-Structured Interviews with Probands and Relatives

| **Category** | **Questions for Probands** | **Questions for Relatives** |
| --- | --- | --- |
| Reasons for participating | - Could you tell us why you decided to participate in this study? - In this study, a genetic counselor offered to contact your relatives to tell them your genetic test results. Did you accept this offer? Why or why not? Did you have specific questions that you needed answered before you made your decision? | - Could you tell us why you decided to participate in this study? - In this study, a genetic counselor contacted you and offered to inform you about your relative’s genetic test results. Did you accept this offer? Why or why not? Did you have specific questions that you needed answered before you made your decision? |
| Experience of intervention | - How would you describe your experience of working with the genetic counselor to contact your family members? - How comfortable were you sharing your relatives’ contact information with the genetic counselor? Would anything have made that process easier for you? - Were there any difficulties with the process (making appointments, communicating with the counselor, etc.)? Did you have any remaining questions after you spoke with the genetic counselor? - How could we improve this relative notification process for you? - How could we improve this relative notification process for your family members? | - What was it like to have the genetic counselor contact you? What was your initial reaction? - Did your relative tell you ahead of time that the study would be contacting you? If so, describe your experience. - Did you decide to learn your relative’s genetic test results? Could you share why or why not? - *[if yes to Q5]* Can you tell us about your experience learning your relative’s genetic test results? What went well? What could have gone better? Did you have any remaining questions after you spoke with the genetic counselor? |
| Benefits / harms of intervention | - What did you do with the genetic test results that were shared with you? (e.g. talked with relatives, talked with primary care doctor, changed my diet, etc.) - Was it important for you to have the information the genetic counselor shared? Why or why not? - Was being in the study helpful or not to you or your family members? In what ways? - Did this relative notification process have any negative effects on you or your family members? In what ways? - Do you have any regrets about choosing to participate in this study? What are those? | - *[if yes to Q5]* What did you do with the information that was shared with you? (e.g., talked with the previously tested relative or other relatives, talked with primary care doctor, made appointment for genetic counseling, got tested, changed my diet, etc.) - Was being in the study helpful or not to you or your family members? In what ways? - *[if yes to Q5]* Was it important for you to have the information the genetic counselor shared? Why or why not? - Did this relative notification process have any negative effects on you or your family members? In what ways? - Do you have any regrets about choosing to participate in this study? What are those? |
| Ethics | - Do you think people (not just in your own family but in any family) should be offered information about their family members’ genetic test results? Why or why not? - Do you think the genetic counselor of the tested family member is the right person to offer this information to relatives? Why or why not? And if not, who do you think the right person is? - Do you have any concerns about your relatives having this information? - What other thoughts do you have about this study or the relative notification process? | - Do you think people (not just in your own family but in any family) should be offered information about their family members’ genetic test results? Why or why not? - Do you think the genetic counselor of the tested family member is the right person to offer this information to relatives? Why or why not? And if not, who do you think the right person is? - What other thoughts do you have about this study or the relative notification process? |
| Alternatives | - If this relative notification process had NOT been available, would you have shared your genetic test results with your relatives directly? If so, how would you have done so? If not, why not? | - If this relative notification process had NOT been available, do you think you would have learned about your relative’s genetic test results? If so, how might you have gotten this information? If not, how would that be for you? |

# References

1. Henrikson NB, Blasi P, Figueroa Gray M, Tiffany BT, Scrol A, Ralston JD, et al. Patient and Family Preferences on Health System-Led Direct Contact for Cascade Screening. J Pers Med. 2021;11(6):538.

2. Makhnoon S, Bowen DJ, Shirts BH, Fullerton SM, Meischke HW, Larson EB, et al. Relationship between genetic knowledge and familial communication of CRC risk and intent to communicate CRCP genetic information: insights from FamilyTalk eMERGE III. Translational behavioral medicine. 2021;11(2):563-72.

3. Bowen DJ, Hay JL, Harris-Wai JN, Meischke H, Burke W. All in the family? Communication of cancer survivors with their families. Fam Cancer. 2017;16(4):597-603.

4. MacDonald DJ, Sarna L, van Servellen G, Bastani R, Giger JN, Weitzel JN. Selection of family members for communication of cancer risk and barriers to this communication before and after genetic cancer risk assessment. Genetics in Medicine. 2007;9(5):275-82.

5. Olson D. FACES IV and the Circumplex Model: Validation Study. Journal of Marital and Family Therapy. 2011;37(1):64-80.

6. Sekhon M, Cartwright M, Francis JJ. Acceptability of healthcare interventions: an overview of reviews and development of a theoretical framework. BMC Health Services Research. 2017;17(1):1-13.

7. Sekhon M, Cartwright M, Francis JJ. Acceptability of health care interventions: A theoretical framework and proposed research agenda. Br J Health Psychol. 2018.

8. Brehaut JC, O'Connor AM, Wood TJ, Hack TF, Siminoff L, Gordon E, et al. Validation of a Decision Regret Scale. Medical Decision Making. 2003;23(4):281-92.

9. Seiffert DJ, McCarthy Veach P, LeRoy B, Guan W, Zierhut H. Beyond medical actionability: Public perceptions of important actions in response to hypothetical genetic testing results. Journal of genetic counseling. 2019;28(2):355-66.
